# Intergenerational Transmission of Overweight and Obesity from Parents to Their Adolescent Offspring – The HUNT Study

**DOI:** 10.1371/journal.pone.0166585

**Published:** 2016-11-16

**Authors:** Marit Næss, Turid Lingaas Holmen, Mette Langaas, Johan Håkon Bjørngaard, Kirsti Kvaløy

**Affiliations:** 1 HUNT Research Centre, Department of Public Health and General Practice, Faculty of Medicine, NTNU—Norwegian University of Science and Technology, Trondheim, Norway; 2 Department of Research and Development, Levanger Hospital, Nord-Trøndelag Health trust, Levanger, Norway; 3 Department of Mathematical Sciences, NTNU—Norwegian University of Science and Technology, Trondheim, Norway; 4 Department of Public Health and General Practice, Faculty of Medicine, NTNU—Norwegian University of Science and Technology, Trondheim, Norway; 5 Forensic Department and Research Centre Brøset, St.Olavs University Hospital Trondheim, Trondheim, Norway; McMaster University, CANADA

## Abstract

**Purpose:**

The main aim of this study was to examine weight associations between parents and offspring at two time points: 1995–97 and 2006–08, taking into account body mass index (BMI) and waist circumference.

**Methods:**

The study included 8425 parent-offspring trios who participated in the population based Health Study of Nord Trøndelag (the HUNT Study), Norway, at either the HUNT2 (1995–97) or the HUNT3 (2006–08) survey. We used linear mixed effects models with siblings clustered within mothers to analyze the associations between 1) parental grouped BMI and offspring BMI z-scores and 2) parental grouped waist circumference and offspring waist circumference z-scores.

**Results:**

Adolescent and adult overweight and obesity were higher in 2006–08 than in 1995–97, with the greatest increase observed in waist circumference. Both mother’s and father’s BMI and waist circumference were strongly associated with corresponding measures in offspring. Compared with both parents being normal weight (BMI <25 kg/m^2^), having two overweight or obese parents (BMI ≥25 kg/m^2^) was associated with a higher offspring BMI z-score of 0.76 (95% CI; 0.65, 0.87) and 0.64 (95% CI; 0.48, 0.80) in daughters, and 0.76 (95% CI; 0.65, 0.87) and 0.69 (95% CI; 0.53, 0.80) in sons, in 1995–97 and 2006–08 respectively. Offspring with one parent being overweight/obese had BMI z-scores of approximately half of offspring with two parents categorized as overweight/obese. The results of the waist circumference based analyses did not differ substantially from the BMI based analyses.

**Conclusions:**

Parental overweight was strongly positively associated with offspring weight both in 1995–97 and 2006–08 where both parents being overweight/obese gave the largest effect. This seemingly stable association, strongly address the importance of public health initiatives towards preventing obesity in parents of both sexes to decrease further obesity expansion in offspring.

## Introduction

The global worry concerning the extent of overweight and obesity among children and adolescents [1, 2] needs drastic preventive measures. While most studies have shown an extensive and stable increase in overweight and obesity over the last decades [3–5], some studies indicate that trends in overweight prevalence differ between time points [6]. Over several decades increases in both body mass index (BMI) and waist circumference have been observed across all age groups and an outstanding rise particularly in waist circumference has been seen in adults [7–9] with the greatest increase detected in the youngest adults (20–29 years). The findings in children have been contradictory. Increase in central obesity in children and adolescents in the period 1977 to 1997 was seen in a UK study [10], while both a US study and data from the Korean National Health and Nutrition Examination Survey (K-NHANES) in Korea, reported the opposite with an abdominal obesity decrease in children and adolescents during the last decade [11, 12]. The understanding of this divergence which is most likely related to the environment or the effects of gene x environment interactions are important to explore. According to twin and adoption studies, there is a strong genetic component in both adult and childhood adiposity and the association between parental and offspring body mass measures are mainly due to common genes rather than shared family environment [13, 14]. Some studies report that both parents`overweight convey obesity risk to offspring [15], while others report a gender-specific risk only by the same-sex parent [16]. There is also evidence to suggest that the intergenerational adiposity association might change over time [17]. Both issues are important matters to fully understand the obesity development and need further investigation.

Both BMI and waist circumference-defined obesity in parents seems to be associated with the corresponding measures of overweight in offspring [18–21]. Most studies reporting these associations, however, have included younger children (age 2–15 years) [20, 22]) and have not addressed these relationships in older adolescent offspring. As mentioned, a greater increase in waist circumference than BMI has been observed over the last decades [3, 8] with apparent corresponding development in parents and offspring. Still we do not know if these associations are equal with regard to general (BMI) and central (waist circumference) adiposity. The aims of this investigation were therefore to determine to what degree parental BMI and waist circumference in full trios were associated with offspring’s corresponding measures in adolescent boys and girls, considering two time-points, 1995–97 and 2006–08. Based on previous literature, we hypothesized that maternal and paternal overweight and obesity affect offspring’s corresponding measures in a gender wise fashion, and that having two overweight parents compared to only one overweight parent will enlarge the effect size.

## Materials and Methods

### Study sample

**Fig 1:**
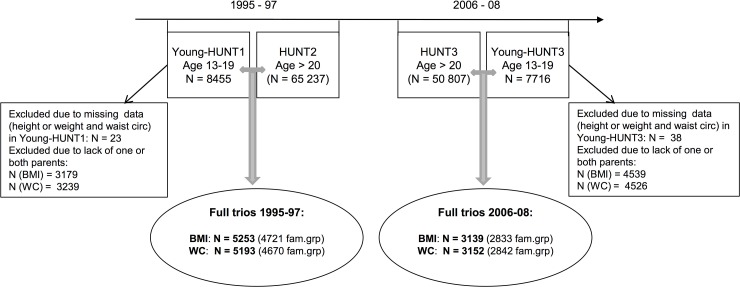
Study flowchart. Number of full parent-offspring trios organized into family groups (fam.grp) due to siblings. Abbreviations: BMI = Body mass index, WC = waist circumference

The Health study of Nord-Trøndelag (The HUNT study) [23–25] is a large population-based health study conducted in the middle of Norway, covering 125 000 participants aged 13 years and above. It consists of three health surveys taken place in 1984–86 (HUNT1), 1995–97 (HUNT2 and Young-HUNT1) and 2006–08 (HUNT3 and Young-HUNT3). Data were collected in all 24 municipalities in the county, and the health examinations were performed by qualified health professional staff in temporarily located sites [23]. The Young-HUNT Study is the adolescent part (13–19 years) of the HUNT Study and was conducted in all the Junior High and High schools in the county. In the Young-HUNT1 survey, 8455 completed a questionnaire and a clinical examination (response rate 83%), while in the Young-HUNT3 survey 7716 participants completed both parts (response rate 74%) (Fig 1). In the adult part of the survey 65 237 individuals participated in 1995–97 (response rate 70%) and 50 807 individuals in 2006–08 (response rate 54%) (Fig 1)[23, 24]. In our study, data from the last two HUNT surveys (1995–97 and 2006–08) were included. We studied associations between parental—and offspring BMI and waist circumference respectively, using cross-sectional data from the two time points. Adolescents who had completed both the questionnaire and the clinical examination and additionally had parents who also had completed both questionnaires and clinical examinations in HUNT at the corresponding time points were included. Only full trios (mother, father and child) were included; yielding a response rate among all participating parents whose adolescent children also participated, of 64% and 56% in 1995–97 and 2006–08 respectively. Individuals without BMI or waist circumference measurements were excluded from the corresponding analyses. The final data sets consisted of 5253 BMI- (4721 family groups due to siblings) and 5193 waist circumference -based (4670 family groups) parent-offspring trios from 1995–97 (HUNT2) and 3139 BMI- (2833 family groups) and 3152 waist circumference-based (2842 family groups) parent-offspring trios from 2006–08 (HUNT3) (Fig 1).

In our sample most of the adolescents were living with both biological parents: 86% in 1995–97 and 85% in 2006–08. The rest lived with only one biological parent (15%) with the other biological parent also attending HUNT.

### Data collection

Data collection included self-reported questionnaires, clinical measurements and structured interviews. Age groups (whole years) for the Young-HUNT participants were defined as age at the nearest birthday; e.g. age 14 years included ≥ 13.5 and < 14.5. Height and weight were measured by trained nurses using the same standardized procedures in both adults and adolescents. The participants wore light clothes and no shoes during the measuring with standardized weight scales and metric bands. Height was measured to the nearest centimeter (cm) and weight to the nearest 0.5 kilogram (kg). Calculation of BMI was weight (kg) divided by squared height (m^2^). BMI status in adults were categorized into normal weight (BMI < 25), overweight (25 ≤ BMI < 30) and obese (BMI ≥ 30) in accordance to World Health Organization (WHO) definitions [7]. Adolescent overweight and obesity characterizations, as provided in guidelines from the International Obesity Task Force (IOTF), were calculated according to cut off points by Cole et al. [26]. Waist circumference was measured to the nearest centimeter applying non-stretchable band horizontally and at the umbilical level after the participants emptied their lungs or midway between the last rib and the iliac cristae if the latter was larger [23, 27]. Waist circumference overweight and obesity cutoff-points in adolescents were based on reference cutoff values (cm) for the 85^th^ and 95^th^ percentiles for Norwegian children according to age and gender [28]. For adults the waist circumference cutoff-points were in accordance with WHO`s guidelines for normal weight (women < 80 cm; men < 94 cm), overweight (women ≥ 80 cm and < 88 cm; men ≥ 94 cm and < 102 cm) and obese (women ≥ 88 cm; men ≥ 102 cm) [7]. For the analysis in our study we combined the overweight and obese category (BMI ≥ 25 kg/m^2^). The same was done for waist circumference (women ≥ 80 cm; men ≥ 94 cm).

#### Ethical approvals

A written informed consent was given by all the participants both in the HUNT and the Young-HUNT studies. For adolescents below the age of 16, parents also gave a written consent. The protocol was approved by the Regional Committee for Ethics in Medical Research, the Data Inspectorate and was in accordance with the Helsinki Declaration.

#### Record linkage

Every citizen in Norway is linked to a unique personal identification number. This enabled the family linkage between the Young-HUNT participants and their biological parents, through the Norwegian Family Register. Likewise, the data on parents’ education was obtained from Statistics Norway (SSB).

### Study variables

Adolescent BMI and waist circumference was transformed into age- and sex-specific z-scores (standard deviations from their means) specific for the two time points. Positive z-scores indicate measures above and negative z-scores indicate measures below the age- and sex-specific mean. S1A and S1B Table present the corresponding age and sex related z-scores and measures in kilograms (BMI) and cm (waist circumference).

Parental BMI and waist circumference categories were further categorized into the following four groups; both parents normal weight, mother overweight and father normal weight, father overweight and mother normal weight, and both parents overweight.

The following potential covariates were investigated in offspring: *pubertal status* (Pubertal Developmental Scale, PDS [29]); (pre pubertal; PD ≤ 1, early pubertal; PD > 1 and ≤ 2, mid-pubertal; PD > 2 and < 3, late puberty; PD ≥ 3 and < 4, and post-pubertal; PD = 4), *consumption of vegetables* (self-reported) by questionnaires and categorized in daily, weekly, seldom and never, as a proxy for diet and *presence of chronic disease* (self-reported, and based on the presence of at least one of the following chronic diseases diagnosed by a medical doctor: asthma, diabetes, migraine or any other illness that lasted longer than 3 months). In parents: *age and education* (three level categorization based on Norwegian Standard Classification of Education (NUS); low = 0–10 years school attendance, medium = 11–14 years school attendance and high > 14 years school attendance [30], *physical activity* (self-reported by questionnaires) in four categories: hard; ≥ 3h hard activity/week, moderate; ≥ 3h light activity and/or 1-2h hard activity/week, low; 1-2h light activity and/or < 1h hard activity/week and inactive; ≤ 1h light activity and no hard activity [31].

### Statistical analyses

Offspring sex stratified analyses were performed separately for the 1995–97 and 2006–08 data. Linear mixed effects models were fitted with siblings clustered within mothers separately for the four strata (girls 1995–97, boys 1995–97, girls 2006–08 and boys 2006–08). Maternal and paternal age was used as covariates, and parents’ overweight as exposure. Parameter estimates with confidence intervals were based on these four main analyses. We also report parameter estimates and confidence intervals for differences between the parental overweight exposure categories, calculated with the aid of linear contrasts from the fitted linear mixed effects models.

In addition to associations between each outcome and exposure between the four strata- four combined models were implemented; For each gender, we fitted combined models for both time points, including interaction terms between time and exposures. For each time point, we fitted combined models for both genders, including interaction terms between gender and exposures and additionally interaction terms with parental age. Precision was measured with 95% confidence intervals.

Stratified analyses with parental education levels as a proxy for socioeconomic status (SES) and adolescent pubertal status (PD score) as a degree of pubertal maturation, were performed. Parents’ education level was represented using the mean of the education levels for both parents, and further classified into two categories; low-medium (≤ 14 years school attendance) and high (> 14 years school attendance). Puberty status was used classified into the two categories less than the number 3 and 3 or above.

To visualize the marginal relationships between maternal and paternal BMI and waist circumference and the offspring’s z-score of BMI and waist circumference, nonparametric regressions using restricted cubic splines were fitted and shown together with 95% confidence intervals. All the statistical analyses were conducted using Stata IC/13.1

## Results

### Descriptions of the cohort

Subject characteristics are summarized in Table 1.

**Table 1:** Descriptive characteristic of offspring and parents.

	Daughters		Sons		Mother		Father	
	1995–97	2006–08	1995–97	2006–08	1995–97	2006–08	1995–97	2006–08
**Number of participants**	2656	1557	2604	1608	5260	3165	5260	3165
**Age, years (SD)**	16.1 (1.8)	16.0 (1.8)	16.0 (1.8)	15.9 (1.7)	42.7 (5.0)	44.7 (5.0)	45.6 (5.6)	47.7 (5.7)
**Height, cm (SD)**	165.7 (6.4)	165.3 (6.4)	174.6 (9.4)	174.3 (9.3)	165.7 (5.6)	166.6 (6.0)	178.6 (6.1)	179.7 (6.1)
**Weight, kg (SD)**	59.0 (10.3)	60.4 (11.4)	65.2 (13.5)	66.9 (14.4)	70.3 (12.3)	73.9 (13.8)	84.6 (11.4)	89.1 (12.8)
**BMI, kg/m2 (SD)**	21.4 (3.3)	22.1 (3.6)	21.2 (3.2)	21.9 (3.6)	25.6 (4.2)	26.6 (4.7)	26.5 (3.2)	27.6 (3.5)
* Underweight/Normal, n (%)	2258 (85%)	1219 (78%)	2189 (84%)	1243 (77%)	2695 (51%)	1375 (43%)	1743 (33%)	722 (23%)
* Overweight, n (%)	326 (12%)	269 (17%)	341 (13%)	279 (17%)	1854 (35%)	1136 (36%)	2827 (54%)	1735 (55%)
* Obese, n (%)	71 (3%)	57 (4%)	73 (3%)	81 (5%)	708 (13%)	648 (20%)	688 (13%)	704 (22%)
**Waist circumference (SD)**	70.4 (7.9)	76.8 (10.2)	75.8 (8.7)	79.0 (10.0)	79,4 (10.5)	89.0 (12.1)	91.2 (8.1)	96.9 (9.4)
** Underweight/Normal, n (%)	1896 (72%)	616 (40%)	1779 (68%)	970 (60%)	2992 (57%)	721 (23%)	3415 (65%)	1184 (37%)
**Overweight, n (%)	390 (15%)	348 (22%)	556 (21%)	332 (21%)	1231 (23%)	847 (27%)	1306 (25%)	1029 (33%)
**Obese, n (%)	350 (13%)	589 (38%)	252 (10%)	300 (19%)	1015 (19%)	1595 (50%)	536 (10%)	951 (30%)
**Pubertal status^A^ (SD)**	3.4 (0.6)	3.3 (0.6)	3.0 (0.7)	2.9 (0.7)				
**Parents Education level** ^**B**^								
Low, n (%)					941 (18%)	359 (11%)	826 (16%)	408 (13%)
Medium, n (%)					2788 (53%)	1480 (47%)	3146 (60%)	1886 (60%)
High, n (%)					1520 (29%)	1317 (42%)	1284 (24%)	865 (27%)
**Parents Physical activity** ^**1**^								
Hard, n (%)					254 (5%)	519 (16%)	611 (12%)	451 (14%)
Moderate, n (%)					1942 (37%)	1246 (39%)	1907 (36%)	1027 (32%)
Low, n (%)					1989 (38%)	602 (19%)	1639 (31%)	672 (21%)
Inactive, n (%)					899 (17%)	179 (6%)	930 (18%)	235 (7%)

Data presented as mean with standard deviation (SD), unless otherwise specified.

^A^Pubertal status (PD score) scale 0.75–4.00: PD ≤ 1; pre pubertal, PD > 1 and ≤ 2; early pubertal, PD > 2 and < 3; mid-pubertal, PD ≥ 3 and < 4; late puberty, PD = 4; post-pubertal.

* BMI (body mass index) based categories in adolescents are age and sex adjusted in accordance with Cole et al. [26], In adults; underweight/normal (BMI < 25), overweight (25 ≤ BMI < 30) and obese (BMI ≥ 30)[7].

** Waist circumference based categories in adolescents are age and sex adjusted in accordance with Brannsether et al. ([28]; In adults: normal weight (women < 80 cm; men < 94 cm), overweight (women ≥ 80 cm and < 88 cm; men ≥ 94 cm and < 102 cm) and obese (women ≥ 88 cm; men ≥ 102 cm) [7].

^1^ Hard ≥ 3h hard activity/week, Moderate ≥ 3h light activity and/or 1-2h hard activity/week, Low = 1-2h light activity and/or < 1h hard activity/week, Inactive ≤ 1h light activity and no hard activity/week.

^B^ Low = 0–10 years school attendance, Medium = 11–14 years school attendance, High > 14 years school attendance.

Mean age (16.0 ± 0.1) in both sexes were similar for adolescents at both time points, while mean age in mothers and fathers were slightly lower in 1995–97 than in 2006–08. Mean weight and waist circumference had increased in all groups from 1996–97 to 2006–08 and overweight and obesity prevalence were higher in 2006–08 than in 1995–97. The greatest increase in obesity was seen in relation to waist circumference (girls; 13% to 38%, boys; 10% to 19%, mothers; 19% to 50% and fathers; 10% to 30%, all in the time periods 1995–97 and 2006–08, respectively).

### Body mass index (BMI) and Waist circumference

A BMI z-score of 0.33 for a 13 years old daughter in 1995–97 corresponds to a weight increase of 2.5 kilograms. Equivalent, a BMI z-score of 0.33 for a 13 years old daughter in 2006–08 corresponds to a weight increase of 3.1 kilograms. See S1A and S1B Table containing z-scores with corresponding anthropometric measures for BMI and waist circumference respectively.

#### BMI based results

Compared with offspring of normal weight parents (BMI < 25), there was an increased offspring BMI if one of the parents was overweight. No considerable difference was identified between mothers and fathers being overweight or between time points. The increase in BMI z-scores when mothers were overweight and fathers normal weight was 0.33 (95% CI: 0.19, 0.46) in both sexes in 1995–97. The association effects were almost the same in 2006–08 with a BMI z-score of 0.37 (95% CI: 0.17, 0.57) for daughters and 0.38 (95% CI: 0.18, 0.58) for sons (Table 2).

**Table 2:** Age adjusted association between parental body mass index (BMI) based overweight and offspring BMI z-score (standard deviations from their means) in 1995–97 and 2006–08.

Covariates	Daughters		Sons	
	1995–97	2006–08	1995–97	2006–08
	BMI z-score (CI)	BMI z-score (CI)	BMI z-score (CI)	BMI z-score (CI)
**Maternal overweight**^**A**^**/paternal normal weight**	0.33 (0.19, 0.46)	0.37 (0.17, 0.57)	0.33 (0.19, 0.46)	0.38 (0.18, 0.58)
**Maternal normal weight/Paternal overweight**^**A**^	0.29 (0.18, 0.40)	0.25 (0.08, 0.42)	0.34 (0.24, 0.45)	0.37 (0.20, 0.53)
**Both parent overweight**	0.76 (0.65, 0.87)	0.64 (0.48, 0.80)	0.76 (0.65, 0.87)	0.69 (0.53, 0.85)
**Maternal age**	-0,02 (-0.04, -0.01)	-0,02 (-0.04, 0.01)	-0,00 (-0.01, 0.01)	-0,01 (-0.02, 0.01)
**Paternal age**	0,02 (0.01, 0.03)	0,01 (-0.00, 0.02)	0,00 (-0.01, 0.01)	0,00 (-0.01, 0.02)

CI = 95% confidence interval

^A^ BMI ≥ 25kg/m^2^

The numbers given are the linear mixed effects regression coefficients between the exposure variables and covariates given as row names and the (age adjusted) BMI z-score of the offspring.

With only fathers being overweight, the BMI z-score in 1995–97 was 0.29 (95% CI: 0.18, 0.40) for daughters and 0.34 (95% CI: 0.24, 0.45) for sons (Table 2). These positive association effects were comparable with the point estimates in 2006–08, yielding a BMI z-score of 0.25 (95% CI: 0.08, 0.42) for girls and 0.37 (95% CI: 0.20, 0.53) for boys at this time point. No statistical interaction between sex and time points (p-value for interaction: 0.33 and 0.55 for daughters and sons, respectively) was found. Additionally, potential interaction between parents BMI and time points were tested, without finding significant differences or major changes in the effect estimates.

**Fig 2:**
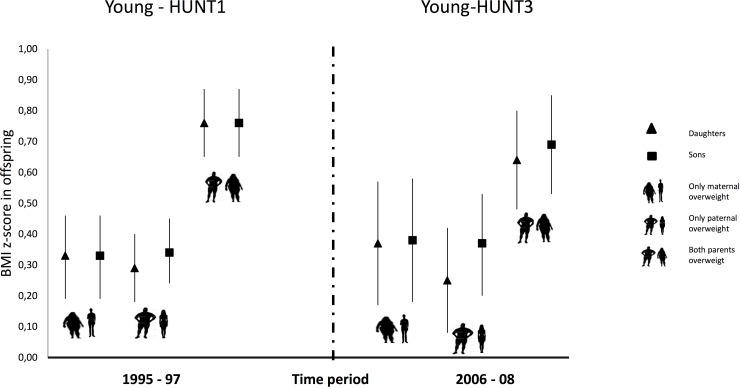
Associations in BMI. Associations between parental BMI-based overweight (BMI ≥25) and offspring`s BMI z- scores at two time points (1995–97 and 2006–08) with both parents being overweight, only mothers being overweight and only fathers being overweight, compared to both parents being normal weight (BMI < 25).

If both parents were overweight the association was substantially stronger compared to only one parent being overweight (Fig 2 and Table 2) observed in all the four main strata (girls and boys in 1995–97 and 2006–08): 0.76 (95% CI: 0.65, 0.87) in both genders in 1995–97, and 0.64 (95% CI: 0.48, 0.80) and 0.69 (95% CI: 0.53, 0.85) in 2006–08, daughters and sons respectively. No statistical interaction between sex and time points was found.

#### Waist circumference based results

**Fig 3:**
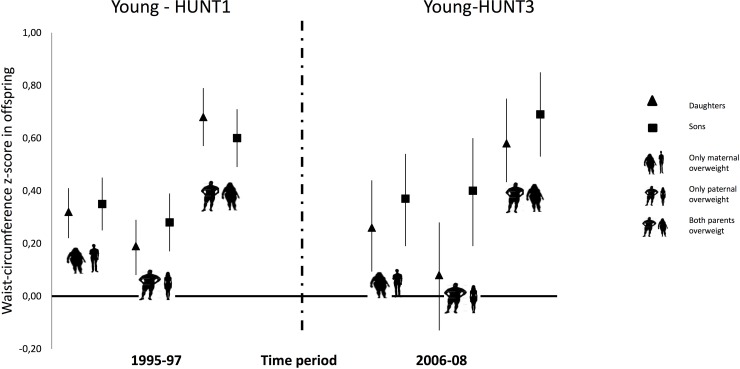
Associations in waist circumference. Associations between parental waist circumference-based overweight (related to WHO`s cut-off level) and offspring`s waist circumference z-scores at two time points (1995–97 and 2006–08), with both parents being overweight, only mothers being overweight and only fathers being overweight, compared to both parents being normal weight.

Associations between parental waist circumference categories and offspring waist circumference z-scores showed a similar pattern to the ones identified for BMI, with the strongest association identified when both parents were overweight; 0.68 (95% CI: 0.57, 0.79) in daughters and 0.60 (95% CI: 0.49–0.71in sons in 1995–97 and 0.58 (95% CI: 0.42, 0.75) and 0.69 (95% CI: 0.53, 0.85) in 2006–08, daughters and sons, respectively (Fig 3, and Table 3).

**Table 3:** Age adjusted association between parental waist circumference based overweight and offspring waist circumference z-score (standard deviations from their means) in 1995–97 and 2006–08.

Covariates	Daughters		Sons	
	1995–97	2006–08	1995–97	2006–08
	WC z-score (CI)	WC z-score (CI)	WC z-score (CI)	WC z-score (CI)
**Maternal overweight**^**A**^**/paternal normal weight**	0.32 (0.22, 0.41)	0.26 (0.08, 0.44)	0.35 (0.25, 0.45)	0.37 (0.19, 0.54)
**Maternal normal weight/Paternal overweight**^**B**^	0.19 (0.08, 0.29)	0.08 (-0.13, 0.28)	0.28 (0.17, 0.39)	0.40 (0.19, 0.60)
**Both parent overweight**	0.68 (0.57, 0.79)	0.58 (0.42, 0.75)	0.60 (0.49, 0.71)	0.69 (0.53, 0.85)
**Maternal age**	-0,01 (-0.02, -0.00)	-0,02 (-0.04, -0.01)	0,00 (-0.01, 0.01)	-0,00 (-0.02, 0.01)
**Paternal age**	0,01 (-0.00, 0.02)	0,01 (-0.00, 0.02)	0,00 (-0.01, 0.01)	-0,00 (-0.01, 0.01)

CI = 95% confidence interval

^A^ cut off value maternal overweight ≥ 80 cm

^B^ cut off value paternal overweight ≥ 94 cm

The numbers given are the linear mixed effects regression coefficients between the exposure variables and covariates given as row names and the (age adjusted) waist circumference z-score of the offspring.

In the group of only mothers being overweight, sons and daughters were equally affected in 1995–97, while in 2006–08 there was a tendency towards sons being more affected than daughters (0.37; 95% CI: 0.19, 0.54 and 0.26; 95% CI: 0.08, 0.29, sons and daughters respectively). For only fathers being overweight, we saw a stronger association in sons compared to daughters at both time points, (Fig 3 and Table 3). However, no sex interaction was seen in the non-stratified model.

#### Supplemental covariate stratifications

For both BMI and waist circumference models, stratified analyses related to parents`education levels were performed. Both groups of only mothers–or only fathers being overweight (BMI) tended to convey greater risks of higher BMI at low education levels compared to higher levels. These associations were strongest for daughters in 2006–08 with both parents being overweight and with low education levels: 0.84 (95% CI: 0.59, 1.08) compared to high education level: 0.39 (95% CI: 0.19, 0.58) (S2 Table). In sons, the association effects were less pronounced.

Based on waist circumference stratified analyses, taking parents`education level into account, showed an association with increased waist circumference in the Young-HUNT1 (1995–97) offspring in low compared to high parental education when overweight occurred in mothers only. We did not see the same relationship in 2006–08. However, when both parents were overweight, the association was somewhat stronger at low level education also in 2006–08 (S3 Table).

Stratified analyses with regards to offspring’s pubertal status (S4 Table) indicated that girls with low puberty scores (PD ≤ 3) tended towards being greater influenced by their parents`overweight (BMI) compared to those with high puberty scores (PD > 3). Adjustment for parental physical activity did not change this association substantially.

Effects of parental waist circumference overweight did not differ substantially between having low—or high puberty score in offspring (S5 Table).

In addition to the analyses already described, we tested the potential influence of factors such as adolescents’ daily intake of vegetables (proxy for diet), physical activity and the presence of chronic disease both for the BMI and the waist circumference models. However, our findings were robust towards these model changes.

Nonparametric regression was used to model the marginal relationship between parental BMI and waist circumference with offspring’s corresponding z-scores (S1 and S2 Figs). A large degree of similarity both related to gender and time points were observed except for the waist circumference relationship between father and sons compared to fathers and daughters in 2006–08, where the slope seemed to be steeper for the father-son than the father–daughter relationship (S2G and S2H Fig).

## Discussion

Parental overweight influenced offspring weight negatively, both in 1995–97 and 2006–08. A substantially increased body mass index (BMI) was found in offspring of parents with BMI-defined overweight compared to normal weight parents. Both parents being overweight approximately doubled the effect size compared to only one parent being overweight. The same associations were seen related to central adiposity measured by waist circumference. Overall the associations were similar for daughters and sons regardless of which parent was overweight. No statistically significant differences in the intergenerational transmission or major changes in the effect estimates were found between the two time points (1995–97 and 2006–08). This is in accordance with findings from several other previous studies [17, 32, 33].

Many previous studies have found that daughters’ overweight is strongly associated with maternal overweight, while overweight in sons are associated with both maternal and paternal overweight [18, 32, 34]. Others have reported stronger association between fathers’ and sons’ BMI [16]. Interestingly and contradictory to this our findings showed that paternal BMI was of same importance for both sons’ and daughters’ BMI.

For waist circumference, on the other hand, we found a tendency towards paternal overweight having less impact on waist circumference in daughters than in sons. This is in partly agreement with findings from the Norwegian HEIA study [18], which showed that maternal overweight measured by waist circumference was associated with corresponding measures in daughters and sons, while paternal overweight was associated with waist circumference overweight only in sons. Generally, possible explanation for this, also stated by others, is that mothers by still being the primary caregivers strongly influence both sons and daughters, while fathers due to behavioral influence, and act as stronger role models for their sons [18, 34]. The weaker coherence between father-daughter compared to father-son relationships identified in our study was further reduced in 2006–08 compared to 1995–97. The total parental overweight still seemed to affect weight in offspring almost equally in both genders, regardless of measuring BMI or waist circumference.

Both parents being overweight, immensely increased offspring`s overweight measured by both BMI and waist circumference. Adjustments for parents’ age did not seem to be of significant importance for the association to offsprings weight. Concerning BMI, our findings are supported in a parent-offspring study with both parents measured when offspring were 11 years old [32]. Increased risk of child obesity when having two overweight parents was also supported by Whitaker et al., although this study included smaller children (age 2 year and up) and young teenagers [20]. Waist circumference associations between parents and offspring seem to be more rarely studied previously than parent–offspring relationship studies based on BMI. As far as we know comparing overweight present both in one and two parents have only been done in one previous Norwegian study [18]; however, children were younger (11 years old) than in our study. Our study showed a substantial increase in BMI and waist circumference between the two time points 1995–97 and 2006–08 both in adults and adolescents. An alarming concern is the increased waist circumference observed in adolescents in the time period investigated, where the number of children defined to be obese has doubled in sons, and tripled in daughters. Our findings with regard to the extensive increased adiposity in the adolescents are supported by the Early Bird study which includes children followed from the age 5 to 15 [19]. Relative to the 1990 UK standards, (a guideline for classifying obesity in children), they saw a substantially greater rise in waist circumference compared to BMI over time with a larger increase in daughters compared to sons. [19]. The increase in central adiposity identified both in adults and adolescents in our study, is mostly supported by previous studies performed on adults, e.g. a study based on the Health Surveys for England in the time period 1993–94 and 2002–03 which also showed a higher increase in waist circumference in women compared to men [21]. The extensive increase in central adiposity, also confirmed in the whole HUNT population [8], seems to be rather general in the population for unknown reasons and one may speculate whether environmental factors or the fact that the sitting time has vastly increased during the last decades can explain part of this adverse development [35]. It could also be a result of a complex genetic/epigenetic interplay which may be due to differential responsiveness to environmental influence [36]. However, the parent-offspring weight relationships identified in our study, shows no change in the associations between the time period 1995–97 and 2006–08, and showed a stable accompanying obesity development.

### Strengths and limitations

The greatest strength of our study is the number of full trios included; 8392 and 8345 based on body mass index (BMI) and waist circumference, respectively. Associations seen in our study are in accordance with previous studies, but the advantage of this study is that it is large and comprises an ethnically homogenous population [23]. Moreover, both BMI and waist circumference were studied and anthropometric measurements were not self-reported, which improves the accuracy and potential bias compared to studies using self-reported values. Also the same protocol for measurements was used in both parents and children. Furthermore, few observations are missing and no imputations have been done. Even so some information could be lost due to the categorization of parental weight instead of using continuous measurements, however, when performing supplemental analyses using parental BMI and waist circumference z-scores as exposures, similar association estimates were identified.

Bias could be inferred due to a reduced response rate from 1995–97 to 2006–08 and comparisons between invited and participating adolescent individuals showed that the non-participants tended to be older, more often boys, and more often attended vocational training compared to academic classes [24]. Some adolescents below the age of 16, failed to return written consents from their parents or guardians, and therefore did not participate in the study. The latter was more noticeable among Young-HUNT3 participants (2006–08) than Young-HUNT1 participants (1995–97) which to some degree may indicate socioeconomic differences between the time points [24]. Another potential weakness which could preclude the judgement of potential effects of shared environment in our study may be that not all parents lived together in the same household. However, this included only about 15% of all trios and it is expected that some of the divorced parents may live part-time together with their children.

## Conclusion

Despite the general strong increase in overweight and obesity during the time investigated, our findings indicated that the degree of transmission of overweight and obesity between parents and their adolescent offspring seem not to have changed within the same time frame (1995–97 and 2006–08). Parental overweight (both BMI- and waist circumference-defined) affected weight in offspring negatively at both time points and the strongest associations were observed when both parents were overweight/obese. In our study, father’s overweight/obesity affected offspring weight similar to mothers, opposed to some previous findings. Parental age was not a significant predictor of offspring weight, and the observed BMI-based associations seemed to be stronger for girls at pre- and early puberty than at late or post puberty. Additionally, the observed associations between parental BMI-defined overweight and offspring`s BMI also seemed to be stronger in adolescents when parental education level was low compared to high. Equivalent associations in offspring`s waist circumference were not observed. The stable accompanying obesity development in parents and their adolescent offspring shown here strongly address the importance of public health initiatives concerning adolescent obesity being directed towards parents—both mothers and fathers.

## Supporting Information

S1 Fig The marginal relationships between parents´BMI and offspring´s z-score of BMI. A visualization of the marginal relationship between parental body mass index (BMI) and the offspring´s z-score of BMI nonparametric regressions, using restricted cubic splines, with a 95% confidence interval.
(DOCX)

S2 Fig The marginal relationships between parents´ waist circumference and offspring´s z-score of waist circumference. A visualization of the marginal relationship between parental waist circumference and the offspring´s z-score of waist circumference nonparametric regressions, using restricted cubic splines, with a 95% confidence interval.
(DOCX)

S1 Table **A–B. A; Age–and sex specific BMI z-score values in correspondence to kilo grams.** BMI deviation from mean = (BMI z-score values* Standard-deviation (SD)) = kg/m^2^ = > Reverse intokilo grams (kg): BMI deviation * Mean height^2^ (m^2^). **B; Age–and sex specific waist circumference z-score in correspondence to centimeter.** Waist circumference deviation from mean (cm) = (WC z-score values* Standard-deviation (SD) centimeters).
(DOCX)

S2 Table Sensitivity analysis in Parental education levels and their association on offspring’s`BMI z-score values. Effect size (from linear mixed effects modelling) in gender offspring BMI z-score at two time points, 1995–97 and 2006–08.
(DOCX)

S3 Table Sensitivity analysis in Parental education levels and their association on offspring’s`waist circumference z-score values. Effect size (from linear mixed effects modelling) in gender offspring waist circumference z-score at two time points, 1995–97 and 2006–08.
(DOCX)

S4 Table Sensitivity analysis in Puberty score levels and their association on offspring’s`BMI z-score values. Effect size (from linear mixed effects modelling) in gender offspring BMI z-score at two time points, 1995–97 and 2006–08.
(DOCX)

S5 Table Sensitivity analysis in Puberty score levels and their association on offspring’s`waist circumference z-score values. Effect size (from linear mixed effects modelling) in gender offspring waist circumference z-score at two time points, 1995–97 and 2006–08.
(DOCX)
