# Supplementary figures and images for: Intergenerational Transmission of Overweight and Obesity from Parents to Their Adolescent Offspring – The HUNT Study
Source: PLoS One. 2016 Nov 16;11(11):e0166585. doi: 10.1371/journal.pone.0166585 (PMC5112991; doi:10.1371/journal.pone.0166585)

**S1 Fig**


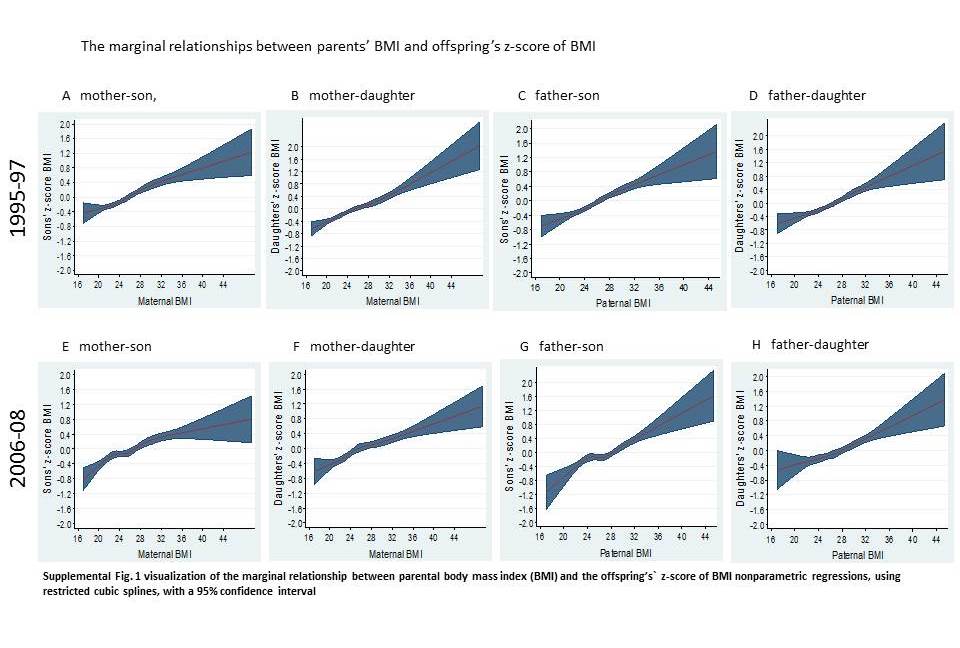

Supplement: S1 Fig — A visualization of the marginal relationship between parental body mass index (BMI) and the offspring´s z-score of BMI nonparametric regressions, using restricted cubic splines, with a 95% confidence interval. (DOCX) [file pone.0166585.s001.docx]

**S2 Fig**


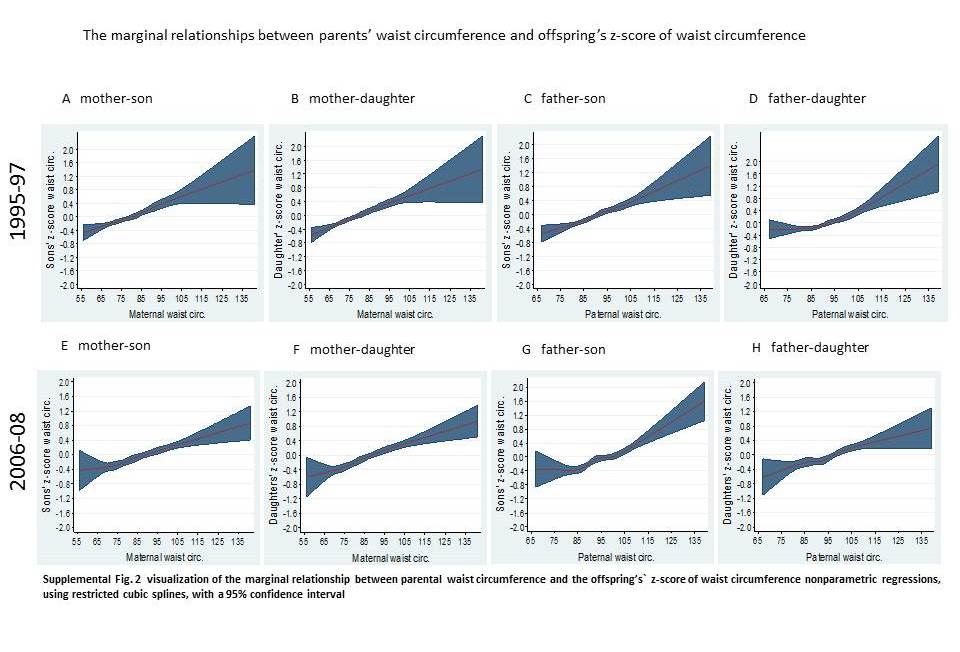

Supplement: S2 Fig — A visualization of the marginal relationship between parental waist circumference and the offspring´s z-score of waist circumference nonparametric regressions, using restricted cubic splines, with a 95% confidence interval. (DOCX) [file pone.0166585.s002.docx]
