# Supplementary material for: Intergenerational Transmission of Overweight and Obesity from Parents to Their Adolescent Offspring – The HUNT Study
Source: PLoS One. 2016 Nov 16;11(11):e0166585. doi: 10.1371/journal.pone.0166585 (PMC5112991; doi:10.1371/journal.pone.0166585)
Supplement: S1 Table — A–B. A; Age–and sex specific BMI z-score values in correspondence to kilo grams. BMI deviation from mean = (BMI z-score values* Standard-deviation (SD)) = kg/m2 = > Reverse intokilo grams (kg): BMI deviation * Mean height2 (m2). B; Age–and sex specific waist circumference z-score in correspondence to centimeter. Waist circumference deviation from mean (cm) = (WC z-score values* Standard-deviation (SD) centimeters). (DOCX) [file pone.0166585.s003.docx]

**S1 Table A
Age – and sex specific BMI z-score values in correspondence to kilo grams**BMI deviation from mean= (BMI z-score values* Standard-deviation (SD)) = kg/m^2^
=> Reverse into kilo grams (kg): BMI deviation * Mean height^2^ (m^2^)
**Daughters YH1 (1995-97)**

| Age (years) | 13 | 14 | 15 | 16 | 17 | 18 | 19 |
| --- | --- | --- | --- | --- | --- | --- | --- |
| Mean height (m) | 1.61 | 1.64 | 1.66 | 1.66 | 1.67 | 1.67 | 1.68 |
| Mean BMI | 19.76 | 20.47 | 20.96 | 21.15 | 22.04 | 22.48 | 23.02 |
| Standard-deviation (SD) | 2.94 | 3.19 | 3.02 | 2.88 | 3.34 | 3.22 | 3.56 |
| BMI z-score values^A^ | 0.33 | 0.33 | 0.33 | 0.33 | 0.33 | 0.33 | 0.33 |
| Deviation into Kilo grams*^A^ | **2.5** | **2.8** | **2.7** | **2.6** | **3.1** | **3.0** | **3.3** |
| BMI z-score values^B^ | 0.29 | 0.29 | 0.29 | 0.29 | 0.29 | 0.29 | 0.29 |
| Deviation into Kilo grams*^B^ | **2.2** | **2.5** | **2.4** | **2.3** | **2.7** | **2.6** | **2.9** |
| BMI z-score values^C^ | 0.76 | 0.76 | 0.76 | 0.76 | 0.76 | 0.76 | 0.76 |
| Deviation into Kilo grams*^C^ | **5.8** | **6.5** | **6.3** | **6.0** | **7.1** | **6.8** | **7.6** |

**Sons YH1 (1995-97)**

| Age (years) | 13 | 14 | 15 | 16 | 17 | 18 | 19 |
| --- | --- | --- | --- | --- | --- | --- | --- |
| Mean height | 1.61 | 1.67 | 1.73 | 1.77 | 1.80 | 1.80 | 1.81 |
| Mean BMI | 19.62 | 19.71 | 20.67 | 21.18 | 21.86 | 22.73 | 22.64 |
| Standard-deviation (SD) | 3.14 | 3.05 | 3.06 | 2.85 | 3.01 | 3.18 | 2.89 |
| BMI z-score values^A^ | 0.33 | 0.33 | 0.33 | 0.33 | 0.33 | 0.33 | 0.33 |
| Deviation into Kilo grams*^A^ | **2.7** | **2.8** | **3.0** | **2.9** | **3.2** | **3.4** | **3.2** |
| BMI z-score values^B^ | 0.34 | 0.34 | 0.34 | 0.34 | 0.34 | 0.34 | 0.34 |
| Deviation into Kilo grams*^B^ | **2.8** | **2.9** | **3.1** | **3.0** | **3.3** | **3.5** | **3.2** |
| BMI z-score values^C^ | 0.76 | 0.76 | 0.76 | 0.76 | 0.76 | 0.76 | 0.76 |
| Deviation into Kilo grams*^C^ | **6.2** | **6.5** | **7.0** | **6.8** | **7.4** | **7.8** | **7.2** |

**Daughters YH3 (2006-08)**

| Age (years) | 13 | 14 | 15 | 16 | 17 | 18 | 19 |
| --- | --- | --- | --- | --- | --- | --- | --- |
| Mean height (m) | 1.62 | 1.64 | 1.64 | 1.66 | 1.66 | 1.67 | 1.68 |
| Mean BMI | 20.15 | 20.66 | 21.39 | 22.39 | 23.14 | 23.27 | 23.28 |
| Standard-deviation (SD) | 3.63 | 3.02 | 3.29 | 3.52 | 3.56 | 3.29 | 3.97 |
| BMI z-score values^A^ | 0.37 | 0.37 | 0.37 | 0.37 | 0.37 | 0.37 | 0.37 |
| Deviation into Kilo grams*^A^ | **3.5** | **3.0** | **3.3** | **3.6** | **3.6** | **3.4** | **4.1** |
| BMI z-score values^B^ | 0.25 | 0.25 | 0.25 | 0.25 | 0.25 | 0.25 | 0.25 |
| Deviation into Kilo grams*^B^ | **2.4** | **2.0** | **2.2** | **2.4** | **2.5** | **2.3** | **2.8** |
| BMI z-score values^C^ | 0.64 | 0.64 | 0.64 | 0.64 | 0.64 | 0.64 | 0.64 |
| Deviation into Kilo grams*^C^ | **6.1** | **5.2** | **5.7** | **6.2** | **6.3** | **5.9** | **7.2** |

**Sons YH3 (2006-08)**

| Age (years) | 13 | 14 | 15 | 16 | 17 | 18 | 19 |
| --- | --- | --- | --- | --- | --- | --- | --- |
| Mean height (m) | 1.61 | 1.67 | 1.73 | 1.76 | 1.79 | 1.80 | 1.81 |
| Mean BMI | 19.72 | 20.62 | 21.31 | 21.72 | 22.89 | 23.19 | 23.77 |
| Standard-deviation (SD) | 2.81 | 3.40 | 3.37 | 3.04 | 3.76 | 3.31 | 3.68 |
| BMI z-score values^A^ | 0.38 | 0.38 | 0.38 | 0.38 | 0.38 | 0.38 | 0.38 |
| Deviation into Kilo grams*^A^ | **2.8** | **3.6** | **3.8** | **3.6** | **4.6** | **4.1** | **4.6** |
| BMI z-score values^B^ | 0.37 | 0.37 | 0.37 | 0.37 | 0.37 | 0.37 | 0.37 |
| Deviation into Kilo grams*^B^ | **2.7** | **3.5** | **3.7** | **3.5** | **4.5** | **4.0** | **4.5** |
| BMI z-score values^C^ | 0.69 | 0.69 | 0.69 | 0.69 | 0.69 | 0.69 | 0.69 |
| Deviation into Kilo grams*^C^ | **5.0** | **6.5** | **7.0** | **6.5** | **8.3** | **7.4** | **8.3** |

^A^ Only maternal overweight, ^B^ Only paternal overweight, ^C^ Both parent overweight
*All values are compared to boys at same age and height where both parents have BMI < 25.

A BMI z-score of 0.33 for a 13 years old daughter in 1995-97 corresponds to a weight increase of 2.5 kilograms. Equivalent, a BMI z-score of 0.33 for a 13 years old daughter in 2006-08 corresponds to a weight increase of 3.1 kilograms.

**S1 Table B
Age – and sex specific waist circumference z-score in correspondence to centimeter**Waist circumference deviation from mean (cm) = (WC z-score values* Standard-deviation (SD) centimeters)

**Daughters YH1 (1995-97)**

| Age (years) | 13 | 14 | 15 | 16 | 17 | 18 | 19 |
| --- | --- | --- | --- | --- | --- | --- | --- |
| Mean waist circumference ,WC (cm) | 66.8 | 68.3 | 69.2 | 70.0 | 72.0 | 72.4 | 74.1 |
| Standard-deviation (SD) | 7.2 | 7.5 | 7.6 | 7.1 | 7.7 | 8.0 | 8.6 |
| WC z-score values^A^ | 0.32 | 0.32 | 0.32 | 0.32 | 0.32 | 0.32 | 0.32 |
| Deviation into centimeters*^A^ | **2.3** | **2.4** | **2.4** | **2.3** | **2.5** | **2.6** | **2.8** |
| WC z-score values^B^ | 0.19 | 0.19 | 0.19 | 0.19 | 0.19 | 0.19 | 0.19 |
| Deviation into centimeter*^B^ | **1.4** | **1.4** | **1.4** | **1.3** | **1.5** | **1.5** | **1.6** |
| WC z-score values^C^ | 0.68 | 0.68 | 0.68 | 0.68 | 0.68 | 0.68 | 0.68 |
| Deviation into centimeters*^C^ | **4.9** | **5.1** | **5.2** | **4.8** | **5.2** | **5.4** | **5.8** |

**Sons YH1 (1995-97)**

| Age (years) | 13 | 14 | 15 | 16 | 17 | 18 | 19 |
| --- | --- | --- | --- | --- | --- | --- | --- |
| Mean waist circumference (WC) (cm) | 70.2 | 72.0 | 74.3 | 76.3 | 77.7 | 79.7 | 79.6 |
| Standard-deviation (SD) | 8.8 | 8.0 | 8.3 | 7.9 | 8.0 | 8.5 | 8.1 |
| WC z-score values^A^ | 0.35 | 0.35 | 0.35 | 0.35 | 0.35 | 0.35 | 0.35 |
| Deviation into centimeters*^A^ | **3.1** | **2.8** | **2.9** | **2.8** | **2.8** | **3.0** | **2.8** |
| WC z-score values^B^ | 0.28 | 0.28 | 0.28 | 0.28 | 0.28 | 0.28 | 0.28 |
| Deviation into centimeter*^B^ | **2.5** | **2.2** | **2.3** | **2.2** | **2.2** | **2.4** | **2.3** |
| WC z-score values^C^ | 0.60 | 0.60 | 0.60 | 0.60 | 0.60 | 0.60 | 0.60 |
| Deviation into centimeters*^C^ | **5.3** | **4.8** | **5.0** | **4.7** | **4.8** | **5.1** | **4.9** |

**Daughters YH3 (2006-08)**

| Age (years) | 13 | 14 | 15 | 16 | 17 | 18 | 19 |
| --- | --- | --- | --- | --- | --- | --- | --- |
| Mean waist circumference (WC) (cm) | 72.9 | 73.2 | 74.7 | 76.6 | 80.1 | 80.5 | 80.4 |
| Standard-deviation (SD) | 10.8 | 8.6 | 9.1 | 10.3 | 9.7 | 9.7 | 11.1 |
| WC z-score values^A^ | 0.26 | 0.26 | 0.26 | 0.26 | 0.26 | 0.26 | 0.26 |
| Deviation into centimeters*^A^ | **2.8** | **2.2** | **2.4** | **2.7** | **2.5** | **2.5** | **2.9** |
| WC z-score values^B^ | 0.08 | 0.08 | 0.08 | 0.08 | 0.08 | 0.08 | 0.08 |
| Deviation into centimeter*^B^ | **0.9** | **0.7** | **0.7** | **0.8** | **0.8** | **0.8** | **0.9** |
| WC z-score values^C^ | 0.58 | 0.58 | 0.58 | 0.58 | 0.58 | 0.58 | 0.58 |
| Deviation into centimeters*^C^ | **6.3** | **5.0** | **5.3** | **6.0** | **5.6** | **5.6** | **6.4** |

**Sons YH3 (2006-08)**

| Age (years) | 13 | 14 | 15 | 16 | 17 | 18 | 19 |
| --- | --- | --- | --- | --- | --- | --- | --- |
| Mean waist circumference ,WC (cm) | 72.5 | 75.6 | 77.8 | 78.1 | 82.7 | 82.6 | 84.4 |
| Standard-deviation (SD) | 8.1 | 9.5 | 9.5 | 8.8 | 10.1 | 9.0 | 10.6 |
| WC z-score values^A^ | 0.37 | 0.37 | 0.37 | 0.37 | 0.37 | 0.37 | 0.37 |
| Deviation into centimeters*^A^ | **3.0** | **3.5** | **3.5** | **3.3** | **3.7** | **3.3** | **3.9** |
| WC z-score values^B^ | 0.40 | 0.40 | 0.40 | 0.40 | 0.40 | 0.40 | 0.40 |
| Deviation into centimeter*^B^ | **3.2** | **3.8** | **3.8** | **3.5** | **4.0** | **3.6** | **4.2** |
| WC z-score values^C^ | 0.69 | 0.69 | 0.69 | 0.69 | 0.69 | 0.69 | 0.69 |
| Deviation into centimeters*^C^ | **5.6** | **6.6** | **6.6** | **6.1** | **7.0** | **6.2** | **7.3** |

^A^ Only maternal overweight, ^B^ Only paternal overweight, ^C^ Both parent overweight
*All values are compared to boys at same age and height where both parents have below cut-off values for overweight related to waist circumference values.

A waist circumference z-score of 0.32 for a 13 years old daughter in 1995-97 corresponds to an increase of 2.3 cm. Equivalent, a waist circumference z-score of 0.32 for a 13 years old daughter in 2006-08 corresponds to an increase of 3.4 cm.
