# Supplementary material for: Intergenerational Transmission of Overweight and Obesity from Parents to Their Adolescent Offspring – The HUNT Study
Source: PLoS One. 2016 Nov 16;11(11):e0166585. doi: 10.1371/journal.pone.0166585 (PMC5112991; doi:10.1371/journal.pone.0166585)
Supplement: S2 Table — Effect size (from linear mixed effects modelling) in gender offspring BMI z-score at two time points, 1995–97 and 2006–08. (DOCX) [file pone.0166585.s004.docx]

**S2 Table
Sensitivity analysis; Parental education levels and their association on offspring’s` BMI z-score values**

**Effect size (from linear mixed effects modelling) in gender offspring BMI z-score at two time points, 1995-97 and 2006-08.**

|  | Daughters | | |  | Sons | | | | |
| --- | --- | --- | --- | --- | --- | --- | --- | --- | --- |
|  | **1995-97** | | **2006-08** | | **1995-97** | | **2006-08** | | |
|  | *BMI z-score (CI)* |  | *BMI z-score (CI)* |  | *BMI z-score (CI)* |  | *BMI z-score (CI)* | |  |
| ***Low education level*** |  |  |  |  |  |  |  | |  |
| **Maternal overweight/paternal normal weight** | 0.37 (0.19, 0.55) |  | 0.47 (0.16, 0.78) |  | 0.29 (0.12, 0.47) |  | 0.51 (0.20, 0.82) | |  |
| **Maternal normal weight/Paternal overweight** | 0.33 (0.17, 0.48) |  | 0.45 (0.18, 0.71) |  | 0.36 (0.21, 0.50) |  | 0.60 (0.34, 0.86) | |  |
| **Both parents overweight** | 0.83 (0.68, 0.98) |  | 0.84 (0.59, 1.08) |  | 0.79 (0.64, 0.93) |  | 0.78 (0.53, 1.02) | |  |
| ***High education level*** |  | | |  |  | | | | |
| **Maternal overweight/paternal normal weight** | 0.27 (0.09, 0.45) |  | 0.27 (0.02, 0.53) |  | 0.39 (0.19, 0.59) |  | 0.26 (-0.01,0.52) |  | |
| **Maternal normal weight/Paternal overweight** | 0.24 (0.18, 0.40) |  | 0.08 (-0.12, 0.28) ns |  | 0.32 (0.16, 0.47) |  | 0.16 (-0.05, 0.36) |  | |
| **Both parents overweight** | 0.58 (0.42, 0.73) |  | 0.39 (0.19, 0.58) |  | 0.64 (0.48, 0.80) |  | 0.61 (0.41, 0.80) |  | |

*CI = 95% confidence interval
Low education level; both parents ≤ 14 years of education (NUS level <2.5 due to NUS2000)*

*High education level; at least one parent > 14 years of education (NUS level ≥ 2.5 due to NUS2000)*
