# Supplementary material for: Intergenerational Transmission of Overweight and Obesity from Parents to Their Adolescent Offspring – The HUNT Study
Source: PLoS One. 2016 Nov 16;11(11):e0166585. doi: 10.1371/journal.pone.0166585 (PMC5112991; doi:10.1371/journal.pone.0166585)
Supplement: S3 Table — Effect size (from linear mixed effects modelling) in gender offspring waist circumference z-score at two time points, 1995–97 and 2006–08. (DOCX) [file pone.0166585.s005.docx]

**S3 Table
Sensitivity analysis; Parental education levels and their association on offspring`s waist circumference z-score values**

**Effect size (from linear mixed effects modelling) in gender offspring WC z-score at two time points, 1995-97 and 2006-08,**

|  | Daughters | | |  | Sons | | | | |
| --- | --- | --- | --- | --- | --- | --- | --- | --- | --- |
|  | **1995-97** | | **2006-08** | | **1995-97** | | **2006-08** | | |
|  | *WC z-score (CI)* |  | *WC z-score (CI)* |  | *WC z-score (CI)* |  | *WC z-score (CI)* | |  |
| ***Low education level*** |  |  |  |  |  |  |  | |  |
| **Maternal overweight/paternal normal weight** | 0.37 (0.25, 0.50) |  | 0.26 (-0.03, 0.55) |  | 0.40 (0.27, 0.53) |  | 0.33 (0.05, 0.60) | |  |
| **Maternal normal weight/Paternal overweight** | 0.23 (0.09, 0.38) |  | 0.09 (-0.26, 0.44) |  | 0.30 (0.15, 0.45) |  | 0.36 (0.03, 0.68) | |  |
| **Both parents overweight** | 0.73 (0.59, 0.87) |  | 0.69 (0.41, 0.96) |  | 0.66 (0.51, 0.80) |  | 0.69 (0.43, 0.94) | |  |
| ***High education level*** |  | | |  |  | | | | |
| **Maternal overweight/paternal normal weight** | 0.19 (0.04, 0.34) |  | 0.26 (0.05, 0.47) |  | 0.23 (0.08, 0.39) |  | 0.40 (0.18, 0.62) |  | |
| **Maternal normal weight/Paternal overweight** | 0.09 (-0.07, 0.26) |  | 0.07 (-0.16, 0.30) |  | 0.25 (0.09, 0.41) |  | 0.42 (0.17, 0.68) |  | |
| **Both parents overweight** | 0.53 (0.35, 0.72) |  | 0.42 (0.23, 0.62) |  | 0.46 (0.29, 0.64) |  | 0.67 (0.46, 0.87) |  | |

*CI = 95% confidence interval
Low education level; Both parents ≤14 years of education (NUS level <2.5 due to NUS2000)*

*High education level; At least one parent > 14 years of education (NUS level ≥ 2.5 due to NUS2000)*
