# Supplementary material for: Intergenerational Transmission of Overweight and Obesity from Parents to Their Adolescent Offspring – The HUNT Study
Source: PLoS One. 2016 Nov 16;11(11):e0166585. doi: 10.1371/journal.pone.0166585 (PMC5112991; doi:10.1371/journal.pone.0166585)
Supplement: S4 Table — Effect size (from linear mixed effects modelling) in gender offspring BMI z-score at two time points, 1995–97 and 2006–08. (DOCX) [file pone.0166585.s006.docx]

**S4 Table
Sensitivity analysis; Puberty score levels and their association on offsprings` BMI z-score values**

**Effect size (from linear mixed effects modelling) in gender offspring BMI z-score at two time points, 1995-97 and 2006-08,**

|  | Daughters | | | |  | | | Sons | | | | |
| --- | --- | --- | --- | --- | --- | --- | --- | --- | --- | --- | --- | --- |
|  | **1995-97** | | | **2006-08** | | | | **1995-97** | | **2006-08** | | |
|  | *BMI z-score (CI)* |  | | *BMI z-score (CI)* | |  | | *BMI z-score (CI)* |  | *BMI z-score (CI)* | |  |
| ***Puberty score ≤ 3*** |  |  | |  | |  | |  |  |  | |  |
| **Maternal overweight/paternal normal weight** | 0.34 (0.10, 0.58) |  | | 0.46 (0.11, 0.81) | |  | | 0.33 (0.14, 0.52) |  | 0.39 (0.11, 0.68) | |  |
| **Maternal normal weight/Paternal overweight** | 0.42 (0.20, 0.63) |  | | 0.32 (0.01, 0.62) | |  | | 0.31 (0.15, 0.46) |  | 0.22 (-0.01, 0.45) | |  |
| **Both parent overweight** | 0.79 (0.57, 1.00) |  | | 0.83 (0.54, 1.12) | |  | | 0.83 (0.67, 0.99) |  | 0.63 (0.41, 0.85) | |  |
| ***Puberty score > 3*** |  | | | |  | | |  | | | | |
| **Maternal overweight/paternal normal weight** | 0.35 (0.19, 0.50) | |  | 0.35 (0.11, 0.60) | | |  | 0.31 (0.13, 0.49) |  | 0.35 (0.06, 0.64) |  | |
| **Maternal normal weight/Paternal overweight** | 0.25 (0.13, 0.38) | |  | 0.20 (-0.00, 0.39) | | |  | 0.37 (0.22, 0.52) |  | 0.48 (0.26, 0.71) |  | |
| **Both parent overweight** | 0.73 (0.61, 0.85) | |  | 0.53 (0.34, 0.71) | | |  | 0.69 (0.54, 0.83) |  | 0.72 (0.51, 0.94) |  | |

*CI = 95% confidence interval
Puberty score ≤ 3; pre – to mid-pubertal*

*Puberty score > 3; late – to post-pubertal*
