# Supplementary material for: Intergenerational Transmission of Overweight and Obesity from Parents to Their Adolescent Offspring – The HUNT Study
Source: PLoS One. 2016 Nov 16;11(11):e0166585. doi: 10.1371/journal.pone.0166585 (PMC5112991; doi:10.1371/journal.pone.0166585)
Supplement: S5 Table — Effect size (from linear mixed effects modelling) in gender offspring waist circumference z-score at two time points, 1995–97 and 2006–08. (DOCX) [file pone.0166585.s007.docx]

**S5 Table
Sensitivity analysis; Puberty score levels and their association on offsprings` waist circumference z-score values**

**Effect size (from linear mixed effects modelling) in gender offspring WC z-score at two time points, 1995-97 and 2006-08,**

|  | Daughters | | | |  | | Sons | | | | |
| --- | --- | --- | --- | --- | --- | --- | --- | --- | --- | --- | --- |
|  | **1995-97** | | | **2006-08** | | | **1995-97** | | **2006-08** | | |
|  | *WC z-score (CI)* | |  | *WC z-score (CI)* | |  | *WC z-score (CI)* |  | *WC z-score (CI)* | |  |
| ***Puberty score ≤ 3*** |  | |  |  | |  |  |  |  | |  |
| **Maternal overweight/paternal normal weight** | 0.27 (0.08, 0.46) | |  | 0.29 (-0.02, 0.60) | |  | 0.40 (0.26, 0.55) |  | 0.36 (0.12, 0.61) | |  |
| **Maternal normal weight/Paternal overweight** | 0.00 (-0.23, 0.23) | |  | 0.09 (-0.17, 0.59) | |  | 0.27 (0.10, 0.43) |  | 0.30 (0.01, 0.59) | |  |
| **Both parent overweight** | 0.57 (0.35, 0.79) | |  | 0.59 (0.29, 0.89) | |  | 0.70 (0.54, 0.86) |  | 0.65 (0.43, 0.88) | |  |
| ***Puberty score > 3*** |  | | | |  | |  | | | | |
| **Maternal overweight/paternal normal weight** | 0.33 (0.22, 0.44) |  | | 0.26 (0.05, 0.47) | |  | 0.31 (0.18, 0.45) |  | 0.35 (0.11, 0.60) |  | |
| **Maternal normal weight/Paternal overweight** | 0.22 (0.09, 0.34) |  | | 0.02 (-0.22, 0.26) | |  | 0.28 (0.13, 0.43) |  | 0.45 (0.17, 0.73) |  | |
| **Both parent overweight** | 0.70 (0.58, 0.83) |  | | 0.57 (0.38, 0.76) | |  | 0.54 (0.24, 0.68) |  | 0.71 (0.48, 0.93) |  | |

*CI = 95% confidence interval
Puberty score ≤ 3; pre – to mid-pubertal*

*Puberty score > 3; late – to post-pubertal*
